# Screening for irradiation vasculopathy by intima-media thickness sonography in head and neck cancer patients

**DOI:** 10.1007/s00405-020-06301-3

**Published:** 2020-09-01

**Authors:** D. Strüder, S. Hellwig, H. Rennau, S. van Bonn, S. P. Schraven, R. Mlynski, G. Hildebrandt, T. Schuldt

**Affiliations:** 1 Department of Oto-Rhino-Laryngology, Head and Neck Surgery “Otto Koerner”, Rostock University Medical Center, Rostock, Germany; 2 Department of Radiotherapy and Radiation Oncology, Rostock University Medical Centre, Rostock, Germany

**Keywords:** Atherosclerosis, Radiotherapy, Stenosis, Ultrasonography, Cardiovascular risk

## Abstract

**Purpose:**

Post-irradiation vasculopathy is a severe form of atherosclerosis and affects the prognosis of head and neck cancer survivors. Sonographic intima-media thickness (IMT) precedes stenosis, plaque formation, and cerebrovascular events. Therefore, IMT may be a valuable screening marker for post-irradiation toxicity. However, the critical irradiation dose and the onset of IMT increase remain unclear.

**Methods:**

The cross-sectional study analysed the carotid artery IMT in 96 irradiated patients and 41 controls regarding irradiation dose, post-irradiation-interval, and cardiovascular risk factors. Distinct irradiation doses to the tumour side and the contralateral hemineck enabled detection of dose depended effects within one patient and control of risk factors.

**Results:**

Radiotherapy caused a dose-dependent increase in IMT. The toxicity did not have saturation effects for > 60 Gy. The IMT increase occurred in short-term following radiotherapy and the risk for a pathological value (> 0.9 mm) rose significantly. The correlation between IMT and radiotherapy was comparable to established cardiovascular risk factors.

**Conclusion:**

Radiotherapists should consider the additional toxicity of high doses for non-metastatic head and neck cancer. If neck metastases require radiotherapy with boost, IMT measurement is suitable for early detection of carotid artery damage.

## Introduction

Radiotherapy is an integral part of head and neck squamous cell cancer (HNSCC) therapy. In particular, for human papillomavirus-related oropharyngeal cancer, high dose radiotherapy (RT) enables 5-year overall survival rates of up to 95% [1–4]. However, the improving outcome and lack of dose de-escalation strategies put the patients at risk for long-term RT-related complications [5, 6]. Post-RT vasculopathy is a common side-effect causing fibrosis (damage to vasa vasorum), platelet aggregation (damage to endothelium), and foam-cell formation (inflammation) [7–10]. Post-RT vasculopathy results in a severe form of atherosclerosis and significantly increases the cerebrovascular risk [7, 8, 10–14]. Carotid artery stenosis results in 18–40% and the relative risk of stroke increases by five compared to healthy controls and non-irradiated cancer patients [5, 10, 11, 14].

To stratify the risk of stroke in irradiated patients, the intima-media thickness (IMT) may be a valuable biomarker [7, 15]. Ultrasonographic IMT is an early indicator of carotid artery damage and accessible during routine cancer-aftercare [7, 10, 12]. The European society of cardiology considers an IMT > 0.9 mm as pathological. The predictive value for cerebrovascular events is established and rises by 13–18% with a 0.1 mm IMT increase [5, 15, 16]. Combining IMT measurement with cardiovascular risk scores (e.g. Framingham Risk Score) may improve the prevention of severe adverse complications after neck RT [5].

Previous research generated robust evidence for increased carotid artery IMT and stenosis following neck RT [6, 12, 17–28]. Gianicolo et al. found a linear correlation between IMT and RT dose between 20 and 70 Gy. Most other studies reported the critical dose below 40 Gy and saturation above [6, 13, 20]. However, a firm RT-dose [Gy] response correlation is not established and the critical dose remains unclear [29]. 50–60 Gy RT with a boost to the gross tumour and the involved lymph nodes is the standard procedure [30]. Therefore, research needs to determine whether the boost effects add toxicity or saturate at high doses.

Pathogenesis of RT vasculopathy includes acute effects such as platelet aggregation and inflammation followed by long-term effects such as fibrosis [7, 8, 10]. Stenosis emerges years after treatment, but the onset of the IMT change remains controversial. Dorrestejn et al. described an increase at the earliest 10 years after RT (while IMT was higher in non-irradiated carotid arteries during the first 10 years) [18]. Another prospective study found the IMT-increase 4 to 9 years after RT [19]. On the contrary, IMT increased 6 weeks after RT in two longitudinal studies compared to the baseline before RT [17, 24]. Further studies reported IMT increases 1 to 3 years after RT [12, 21, 22, 25]. In summary, the onset of IMT increase, and the value for the screening of carotid artery damage remain unclear.

Given the claims made by previous research, we assert that carotid artery IMT increases after neck RT in the short-term. Therefore, we analysed the carotid artery IMT of the tumour side and the contralateral side after bilateral RT with a unilateral boost. The onset of IMT increase was examined by ultrasonography in post-RT intervals from 6 months to 15 years.

## Materials and methods

The cross-sectional study compared carotid artery IMT in irradiated (± boost on tumour side) and non-irradiated head and neck cancer patients at unique time points. Ultrasonographic IMT was measured during tumour after-care. Then, IMT was correlated with RT (dose, boost, post-RT interval) and clinical data from chart review (adjuvant treatment, cardiovascular risk factors).

### Patients

Patients were approached during cancer aftercare at a tertiary university hospital with certified head and neck cancer centre. Written informed consent for participation and publication was obtained from all participants following the local ethics committee (A 2013–0073) according to the 1964 Declaration of Helsinki. Consecutive patients were approached during cancer aftercare and included after RT of a head and neck squamous cell cancer (oral cavity, oropharynx, hypopharynx, and larynx) at the Department of radiotherapy and radiation oncology between 2001 and 2013. Head and neck cancer patients that underwent surgery only and random (non-cancer) otorhinolaryngology patients formed the control group. Exclusion criteria were recurrent disease and prior neck surgery (not-disease specific, e.g. cyst-/lymph node excision, carotid artery surgery). Clinical data were assessed by chart review and comprised demographics, body mass index, smoking habits, tumour TNM, and treatment regimen.

### Radiation protocol

RT was performed for 6 weeks (range 3–7,5 weeks) by intensity-modulated RT, 2D, or 3D conformal RT. The following parameters were recorded from chart review and RT protocol: technique, dose, sidedness (bilateral or unilateral), schedule [conventional (1.8–2.0 Gy/fraction, 5 treatments/week), accelerated (1.8–2.0 Gy/fraction, > 5 treatments/week), hyperfractionation (< 1.25 Gy/fraction, 10 treatments/week), or hypofractionation (> 2 Gy/fraction once daily)]. Based on the planning computer tomography, the dose to the distal 2 cm of the common carotid artery was calculated by OnCentra^®^ 4.3. The post-RT interval was defined as the time between the end of RT therapy and IMT measurement.

### Ultrasonography

**Fig. 1:**
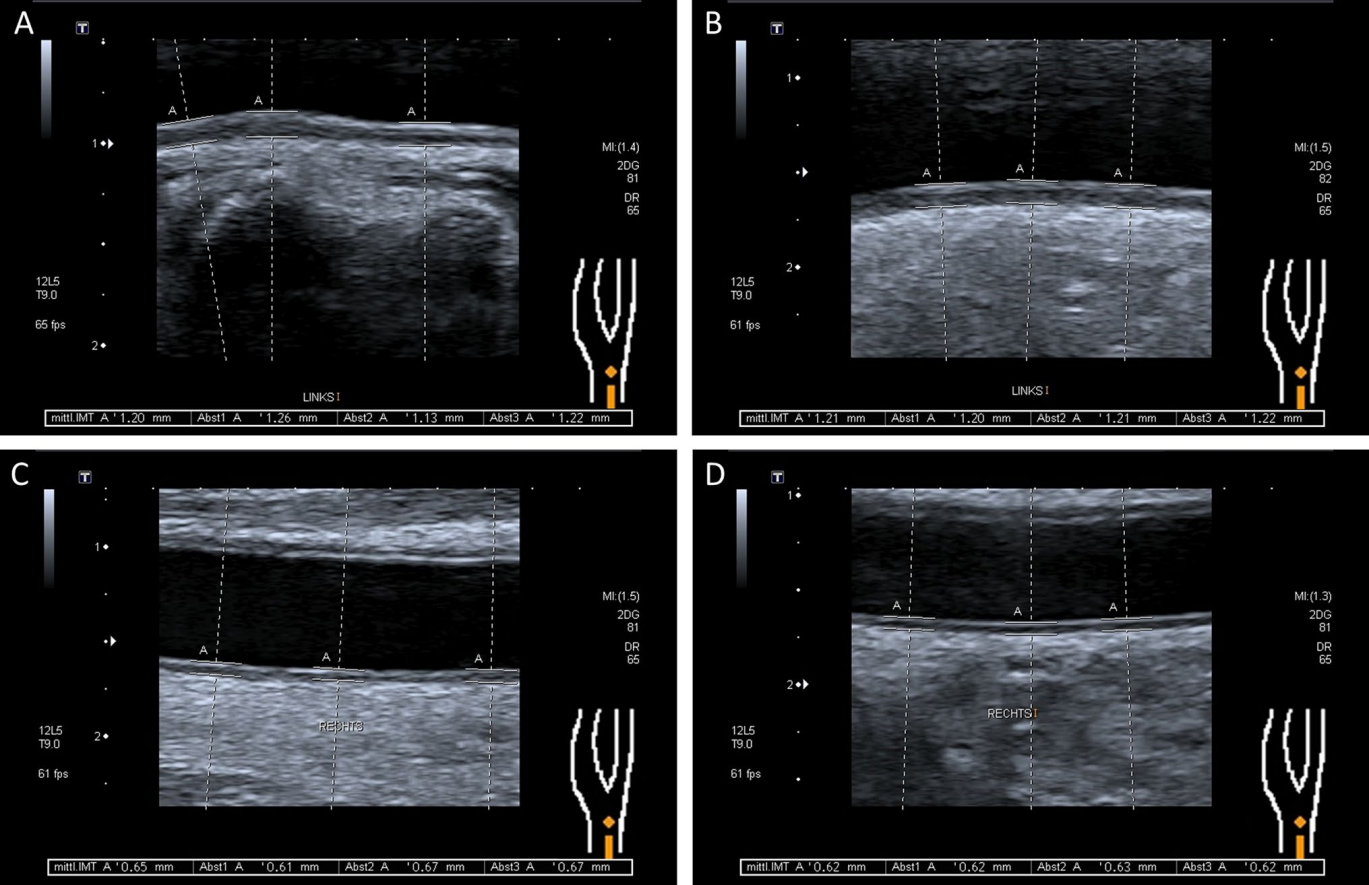
Measurement of the carotid artery intima-media thickness. Figure 1 shows longitudinal B-mode ultrasound images of the common carotid artery (CCA) proximal to the carotid bulb. Three measurements were performed to calculate the mean intima-media thickness (IMT). **a**, **b** Illustrate increased IMT values of 1.20 mm and 1.21 mm following surgery and additive irradiation therapy with a dose of 64 and 63.5 Gy to the respective carotid artery (**a** laryngeal cancer: pT3 pN0 cM0 R0_(close margins)_; **b** oropharyngeal cancer: pT2 pN2c cM0 R2). In contrast, inconspicuous IMT values of 0.65 mm are shown after surgery and adjuvant irradiation with 56 Gy (**c** oropharyngeal cancer: pT2 pN2b cM0 R0) and surgery only (**d** laryngeal cancer: pT1 cN0 cM0 R0)

The ultrasonography was carried out using a Toshiba Xario-Typ SSA-660A (Toshiba, Minato, Japan) with a linear transducer (PLT-805AT) in B-mode with a mean frequency of 9 MHz. The IMT was defined as the distance between the echogenic line representing the blood-intima interface and the echogenic line representing the media-adventitia interface. The IMT was measured on the posterior wall of the distal 2 cm of the common carotid artery in the longitudinal plane, using an anterolateral approach with the transducer head perpendicular to the vessel [31]. Three IMT measurements were performed on twofold magnified still images by two examiners (TS, SH) (Fig. 1). The respective means were used for analysis. Clinical data were not available during IMT measurement.

### Statistics

All statistical tests were selected before data collection. Quantitative variables were expressed as means (± standard deviations) and medians (including ranges). Data were summarized by descriptive statistics and the D’Agostino–Pearson normality test was performed. With parametric data, paired samples (tumour side vs. contralateral side) were compared using (multiple) *t *tests (Holm–Sidak’s posthoc-test). Multiple comparisons were performed by One Way Anova (Tukey) or Welch’s ANOVA for unequal variances (Dunnet’s T3 posthoc-test). With non-parametric data, two groups were compared by Mann–Whitney test and Wilcoxon’s test if paired; multiple comparisons were performed by Kruskal–Wallis-test (Dunn’s posthoc-test)*.* Relationships between variables were analysed by Spearman correlation and simple/multiple linear regression models (least squares). Risk ratios were calculated by Fisher’s exact test. Results were considered significant for *p* < 0.05. All calculations were performed with GraphPad Prism8 (GraphPad Software, San Diego, US).

## Results

The study examined the impact of head and neck RT on carotid artery IMT. The IMT increased after RT (compared to unirradiated carotid arteries), and further increased after boost RT to the tumour side (compared to the contralateral side). The IMT increase occurred in short-term following radiotherapy and the risk for a pathological value (> 0.9 mm) rose significantly. The correlation between IMT and radiotherapy was comparable to established cardiovascular risk factors.

The patients were treated with diverse strategies (RT ± chemotherapy/ ± surgery; surgery only) and examined at different time points after therapy. 117 consecutive head and neck cancer patients were recruited: 96 patients in the radiotherapy group, 21 patients in the surgery only group, and 20 healthy participants in the control group. Participants were predominantly > 60-year-old male smokers. Age, sex, and cardiovascular risk factors, which may interfere with intima-media thickness, did not differ between the groups (except for fewer smokers in the control group). Ultrasonography was performed 1 month–12 years (mean 3 years) after RT/surgery [Table 1].

**Table 1:** Characteristics of the 137 study participants

Group characteristics	Irradiation *n *= 96	Surgery only *n *= 21	Healthy control *n *= 20
Female Male	16 (17%) 80 (83%)	4 (19%) 17 (81%)	5 (25%) 15 (75%)
Age [years]	62.82 ±11.04	60.09 ±12.85	61.45 ±9.90
BMI [kg/m²]	24.56 ±3.32	26.05 ±4.50	26.62 ±3.33
Tabacco [PY] 0–9 PY 10–29 PY ≥30 PY	19.33 ±18.61 38 (40.43%) 33 (34.38%) 23 (24.47%)	17.52 ±18.80 10 (47.62%) 4 (19.04%) 7 (33.33%)	8.45 ±11.16* 16 (80%) 4 (20%) 0 (0%)
Hypertension	41 (42.70%)	8 (38.09%)	4 (20%)
Diabetes mellitus	10 (10.41%)	2 (9.52%)	0 (0%)
Dyslipidaemia	13 (13.54%)	2 (9.52%)	0 (0%)
Cerebrovascular events	23 (23.96%)	1 (4.76%)	0 (0%)

Values depict absolute/relative numbers and mean ±SD. D’Agostino–Pearson normality test and One Way ANOVA (Tukey) were performed (*p < 0.05)

*BMI* body mass index, *PY* pack years

Among the RT group, most patients were treated with a 2 Gy single dose (77/96; e.g. planning target volume 50 Gy and 14 Gy boost). 40/96 irradiated patients had bilateral neck RT (< 5 Gy left/right neck difference); 55/96 received a boost dose to the tumour side hemineck [Table. 2]. The RT dose to the carotid artery was comparable for bilateral RT (62.5 Gy ± 7.9) and the tumour side boost (61.1 Gy ± 9.7). Contralateral to the tumour side, less RT dose was delivered (49.3 Gy ± 15.6, *p* < 0.5). Most irradiated patients were additionally treated with chemotherapy (55/96) and/or surgery, including a neck dissection (65/96).

**Table 2:** Treatment of the 96 irradiation patients

	Mean/*n*	±SD/%
Sidedness		
Bilateral Tumour side boost [>5 Gy]	41/96 55/96	36 64
Dose to CCA [Gy]		
Bilateral Tumour side Contralateral	62.5 61.1 49.3	±7.9 ±9.7 ±15.8*
Tumour side boost [Gy]	15.63	±15.6
Protocol [N]		
1.50 Gy/fraction 1.80 Gy/fraction 2.00 Gy/fraction 2.25 Gy/fraction	6/95 5/95 77/95 7/95	7 5 80 8
Add. chemotherapy [N] Add. surgery [N]	55/95 65/95	57 67767
Post-irradiation interval [years]	3.1	±3.1

Values depict mean ±SD and absolute/relative numbers. D’Agostino–Pearson normality test and One Way ANOVA (Tukey) were performed (*p < 0.05 vs bilateral/tumour side)

*CCA* common carotid artery, *add* additional

**Fig. 2:**
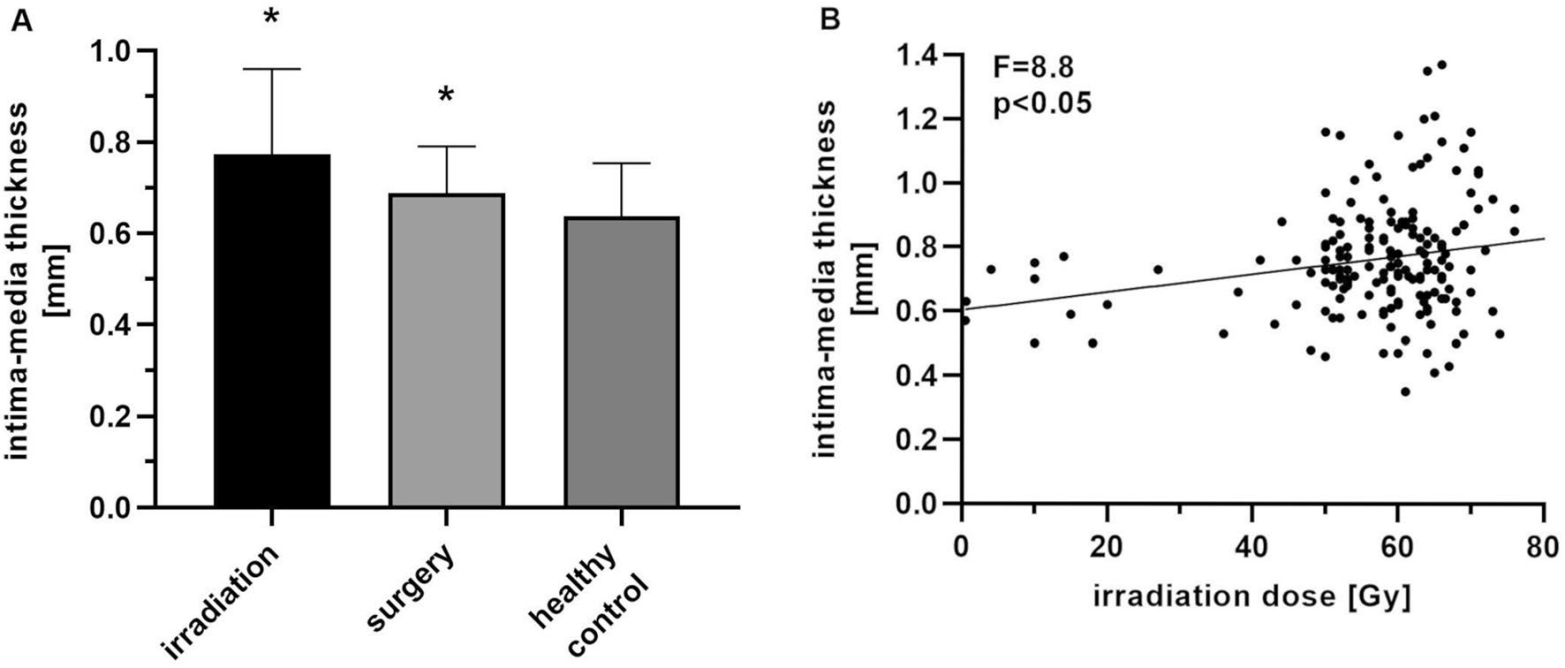
Impact of irradiation on intimamedia thickness. **a** Intima-media thickness of irradiated and non-irradiated carotid arteries. Intima-media thickness was measured after ipsilateral and bilateral neck irradiation (0.76 ± 0.15 mm, *n* = 96). Patients that underwent surgery without irradiation (0.69 ± 0.10 mm, *n* = 21) and healthy participants (0.64 ± 0.11, *n* = 20) served as controls. The D’Agostino–Pearson’s normality test and Welch’s ANOVA were performed (Dunnet’s T3, *p < 0.05 vs. irradiation). **b** Simple linear regression of irradiation dose and intima-media thickness. Patients with unilateral and bilateral (mean of both sides) neck irradiation were included (*n* = 96, *F* = 8.8, *p* < 0.05)

**Fig. 3:**
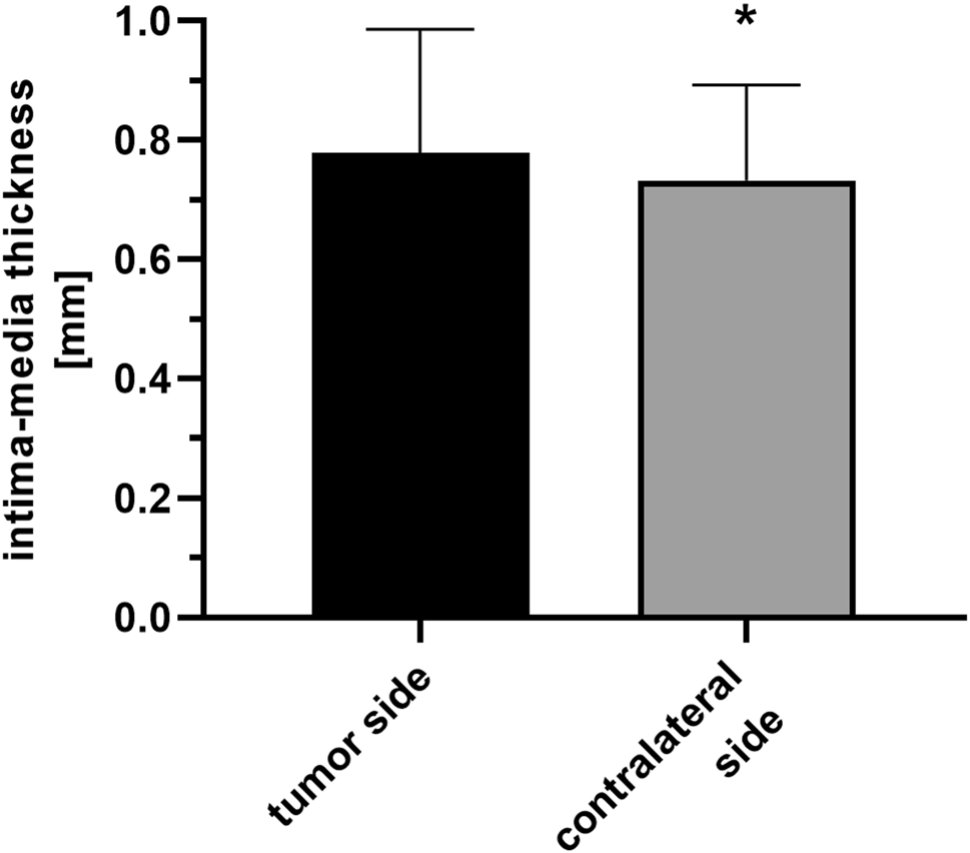
Intima-media thickness increase of tumour hemineck carotid arteries. Intima-media thickness of irradiated carotid arteries with Δ > 5 Gy difference between the tumour and the contralateral side (mean intima-media thickness 0.78 ± 0.20 mm vs. 0.73 ± 0.16 mm). D’Agostino–Pearson’s normality test and paired *t* tests were performed (*n* = 55, *p < 0.05 vs. tumour side)

High dosage RT significantly increased carotid artery IMT. The mean IMT following RT was 13% higher than the surgery only group and 20% higher than the healthy control group (RT: 0.77 ± 0.18 mm; surgery: 0.69 ± 0.10 mm, control: 0.64 ± 0.12 mm, Welch’s ANOVA, Dunnet’s T3, *p < 0.05) (Fig. 1). A simple linear regression was calculated to predict the IMT based on RT dose (Fig. 2). A significant regression equation was found (*Y* = 0,002,801 × *X* + 0,6034, *F* = 8.8, *p* < 0.05), with R^2^ of 0.05. The difference in dose of the tumour side and the contralateral side was > 5 Gy for 61 irradiated patients. Among them, the IMT of the tumour side carotid artery—treated with additional 15.6 ± 15.6 Gy—was 7% higher than the contralateral IMT (0.78 mm ± 0.20 vs. 0.73 mm ± 0.15; paired *t* test, *p *< 0.05) (Fig. 3).

**Fig. 4:**
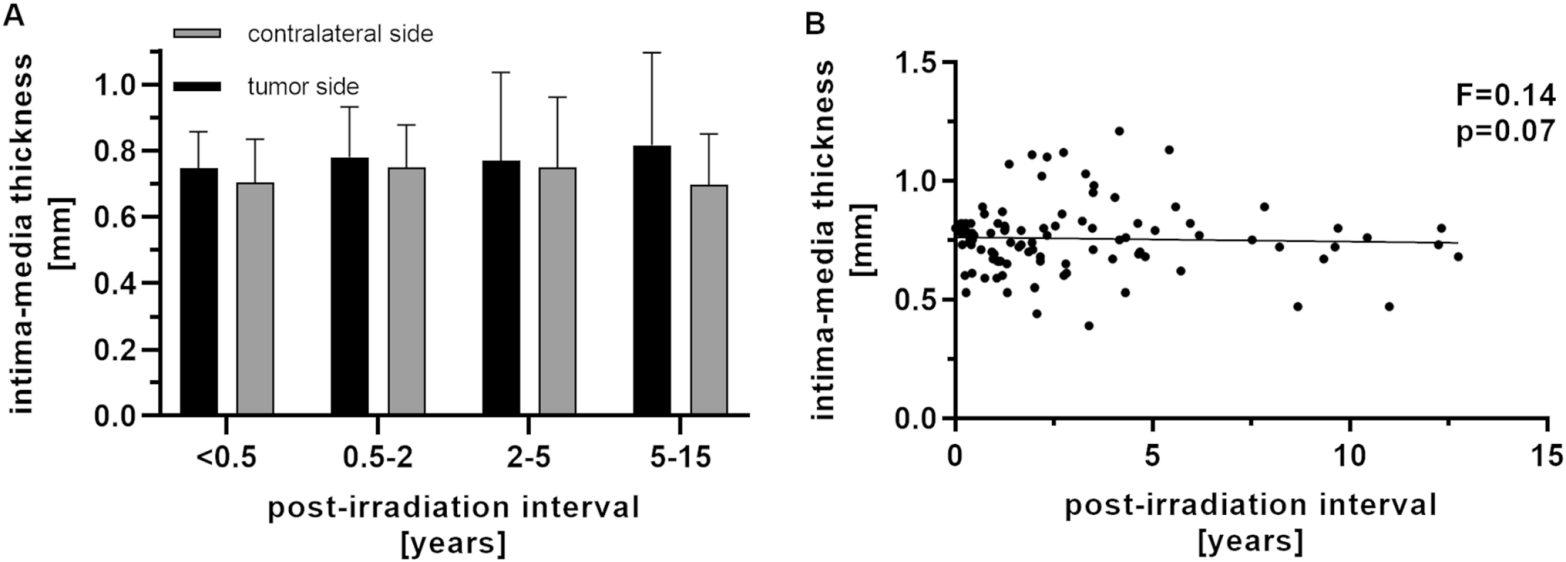
Impact of post-irradiation interval on intima-media thickness. **a** Patients with unilateral irradiation boost were grouped according to the post-irradiation interval of < 0.5, 0.5–2, 2–5 and > 5 years (*n* = 11, 23, 15, 6). The tumour side and contralateral side were compared by multiple *t *tests (Holm–Sidak correction, *p* > 0.05, respectively). **b** Simple linear regression of post-irradiation interval and intima-media thickness (*Y* = − 0.001850 × X + 0.622, *N* = 96, *F* = .14, *p* > 0.05)

Following RT, the IMT increased early and independently from the length of the post-RT interval. Early ultrasonography—within 6 months after RT—showed a slight increase in IMT for the tumour side (0.75 ± 0.10 mm) compared to the contralateral side (0.70 ± 0.13 mm, multiple *t *tests, *p* > 0.05) (Fig. 4). The difference was maintained over 10 years: 0.5–2 years (tumour side 0.78 ± 0.15 mm /contralateral 0.75 ± 0.13 mm, *p* > 0.05); 2–5 years (tumour side 0.77 ± 0.26 mm/contralateral 0.75 ± 0.21 mm, *p* > 0.05); > 5 years (tumour side 0.81 ± 0.28 mm/contralateral 0.70 ± 0.15 mm). However, the post-RT interval showed no correlation with IMT of irradiated carotid arteries (simple linear regression, *Y* = − 0,001,850 × *X* + 0,7622, *F* = 0.14, *p* > 0.05).

RT—but not chemotherapy and neck dissection—increased the risk for pathological IMT swelling. The relative risk for an IMT > 0.9 mm after irradiation was 1.19 (95% CI 1.04–1.34), while the attributable risk was 6.47 (95% CI 3.42–17.6). While RT correlated with IMT (*r* = 0.31, *p* < 0.05), additional chemotherapy and neck dissection showed no correlation. Simultaneous platinum-based chemotherapy was performed in 57% of the irradiated patients. The mean IMT was the same for RT only and chemoradiation (0.76 ± 0.16 mm, *n* = 40 vs. 0.75 ± 0.14 mm, *n* = 55, *t *test *p* > 0.05) and chemotherapy was not correlated with IMT (*r* = 0.14). 67% of the irradiated patients underwent a neck dissection. The IMT increased after RT and neck dissection compared to neck dissection only (0.75 ± 0.16 mm, *n* = 65 vs. 0.70 ± 0.09 mm, *n* = 12; *t* test *p* > 0.05). Such as chemotherapy, neck dissection was not correlated with IMT (*r* = 0.01). Analysis of IMT and cardiovascular (Framingham) risk factors revealed the strongest correlation for age (*r* = 0.50), smoking (*r* = 0.22), dyslipaemia (*r* = 0.22) and diabetes mellitus (*r* = 0.22). Only the correlation between IMT and age (*r* = 0.50) was stronger than for RT (*r* = 0.31). Further risk factors (male gender, adipositas, hypertension, alcohol abuse and prior cardiovascular events) did not correlate with IMT (Table. 3). In a multiple linear regression, the addition of RT to Framingham cardiovascular risk factors improved the predictive value of IMT increase: A multiple linear regression was calculated to predict IMT based on age, gender, dyslipaemia, smoking, and hypertension. A significant regression equation was found [*F* (6, 127) = 9.49, *p* < 0.05], with R^2^ of 0.3. Addition of RT to the independent variables improved the correlation (*F* 7, 126) = 9.75, *p* < 0.05), with R^2^ of 0.35. Age, smoking, dyslipaemia, and RT were significant predictors of IMT.

**Table 3:** Correlation between intima-media thickness and treatment/cardiovascular risk factors

Factor	Spearman *r*	95% CI
Irradiation	0.31*	0.15 to 0.46
Neck dissection	0.02	− 0.15 to 0.19
Chemotherapy	0.14	− 0.03 to 0.30
Age	0.50*	0.36 to 0.62
Gender	− 0.01	− 0.18 to 0.16
Adipositas	− 0.09	− 0.26 to 0.09
Diabetes mellitus	0.21*	0.04 to 0.37
Dyslipidaemia	0.22*	0.05 to 0.38
Hypertension	0.18	0.01 to 0.34
Smoking	0.22*	0.05 to 0.38
Alcohol abuse	0.16	− 0.01 to 0.33
Cardiovascular events	0.11	− 0.06 to 0.29

Risk factors defined as male gender, adipositas (> 30 kg/m²), smoking (> 10 pack years) alcohol abuse (> 1 standard drink/d), diabetes mellitus, dyslipidaemia, hypertension, cardiovascular events (all anamnestic). D’Agostino–Pearson normality test and Spearman correlation were performed (*n* = 137, *p < 0.05)

*CI *confidence interval

In summary, RT increased the IMT of carotid arteries and a tumour side boost (> 5 Gy) added toxicity compared to the contralateral side. Following RT, the IMT increased in short-term and the risk for a pathological value (> 0.9 mm) rose significantly. Unlike RT, chemotherapy and neck dissection did not correlate with IMT.

## Discussion

Head and neck cancer RT cause carotid artery damage that results in increased IMT, stenosis and enhanced risk of stroke [5, 8, 10, 11, 14]. However, the effect of the tumour side boost (> 60 Gy) remains unclear. This study examined the IMT in head and neck cancer patients following bilateral RT with a unilateral boost. RT without boost served as internal control and non-irradiated patients as external control. This is the first study to show the prominent effect of RT with boost: RT caused a dose-dependent increase of IMT, and RT with > 60 Gy enhanced the risk of a pathological IMT.

The finding of RT-induced carotid artery IMT increase and stenosis has been reported previously. However, research focused on IMT or stenosis after RT compared to unirradiated control groups, unirradiated heminecks, and longitudinal follow-up after RT. Because of these designs, the dose–response relationship and the impact of the tumour side boost remain controversial. Some studies described unaffected carotid arteries after RT (for at least 10 years) [18, 20, 21] Noticeably, these studies included younger patients with RT-protocols for lymphomas and parotid tumours. These protocols (~ 75% 50–60 Gy) may be more comparable to the contralateral hemineck dose of head and neck cancer patients (no IMT differences in our study). Most head and neck cancer studies described an IMT increase after RT compared to pre-RT measurements and healthy control groups [6, 17, 22–25, 27]. Gujral et al. (60% > 60 Gy) found an IMT increase in irradiated heminecks (53 ± 13 Gy) compared to the non-irradiated contralateral neck (1.9 ± 3.7 Gy) [12]. In line, Brown et al. reported increased carotid artery stenosis after unilateral RT (59 (45–70) Gy vs.10 (0–28) Gy) without statistical significance (*n* = 44). Our study first examined the differential effects of bilateral irradiation with unilateral boost: The most important finding is the additional IMT increase of 7% due to the tumour side boost (15.6 Gy). In contrast to the tumour side, the IMT of the contralateral irradiated side was not higher than the surgery only and control groups’ IMT. Therefore, this study adds evidence to RT dose-related carotid artery damage and the toxicity of the tumour side boost.

Another controversial issue is the onset and progression of IMT increase. According to our results, the increase in IMT occurs early after RT and remains for up to 15 years. The onset of the IMT-increase must be differentiated from carotid artery stenosis, which emerges years after treatment [6, 9, 13, 26, 27]. Therefore, stenosis is not suitable for early detection of post-RT vessel damage. However, IMT responds earlier, but the timing remains controversial: Dorrestejn et al. and Wilders et al. did not find an increase of IMT within four to 10 years. In this setting, IMT measurement is not meaningful, because the pathological state of stenosis already emerges. However, most studies are in line with our findings reporting an early IMT increase within intervals of weeks or months after RT [12, 17, 21, 22, 24]. Toprak et al. and Pereira et al. found increased IMT measures as soon as 6 weeks after radiotherapy [17, 24]. This increase might be temporary due to the subacute inflammation, but the difference in IMT remained the same after 6 months [24]. In our study, the IMT remained steadily increased for years. This finding is likely explained by the pathogenesis: Acute inflammation and platelet aggregation are continuously followed by long-term foam cell formation and fibrosis. Therefore, acute processes cause an IMT increase that is maintained by chronic inflammation. Hence, carotid artery IMT appears to be a suitable surrogate for early detection of RT-induced carotid artery damage [5, 8].

The increase of IMT may be an early indicator of the doubled risk for cerebrovascular events in irradiated head and neck cancer patients [6, 15]. An IMT increase of 0.1 mm is supposed to enhance the risk of stroke by 13–18% [5, 10, 14–16]. The European Society of Cardiology considers the IMT of > 0.9 mm as pathological. The risk for a pathological IMT after RT was significantly increased: 18% of the RT-group vs. 2.5% of the control group. 6.47 patients (95% CI 3.42–17.6) had to be irradiated to cause one IMT of > 0.9. Therefore, the RT-effect is likely to be clinically relevant and may result in future cerebrovascular events.

The clinical relevance is supported by the dose-related correlation of RT and IMT. The correlation was stronger than most factors (except age) in cardiovascular risk assessment scores (e.g. smoking, diabetes mellitus, dyslipaemia in Framingham risk score). Therefore, adding RT to cardiovascular risk scores improves the predictive value for carotid artery damage (multivariate analysis). This is in line with previous research, which identified RT as the dominant factor for cardiovascular events in irradiated head and neck cancer patients [13, 26, 32]. With one dominant factor, standard cardiovascular scores lack predictive value and adding RT and/or IMT may be beneficial for risk stratification [15]. In contrast to RT, surgery and chemotherapy did not affect the IMT in our study. In line with our study, previous research neither found evidence for severe carotid artery toxicity for chemotherapy and neck dissection [6, 12, 20, 27].

Our cross-sectional study had several limitations: The major limitation is the lack of baseline IMT values before RT. Therefore, other factors might have contributed to the IMT increase in the RT group. However, IMT increased in irradiated patients only and the effect was stronger on the tumour side hemineck. Within the group of 55 patients with a mean 15 Gy difference between the tumour and the contralateral side (mean IMT 0.78 ± 0.20 mm vs. 0.73 ± 0.16 mm), the contralateral side can be considered an internal baseline for the tumour side boost’s effects. Therefore, we could attribute the IMT increase (primarily) to the RT dose.

A second limitation is the unstandardized post-radiation interval (consecutive patients). Because of the after-care algorithms, most patients presented within 5 years post-RT. However, this distribution focused on the dynamic phase of IMT change and might have been beneficial for the verification as an early biomarker. Additionally, RT protocols differed depending on whether the patients had unilateral, bilateral and no neck metastases. Also, a variety of RT techniques were adapted throughout the study period between 2000 and 2013. To account for these limitations, the exact RT dose delivered to the respective carotid arteries was calculated from the target fields. During the study period, most patients had bilateral RT (only 5/96 patients received < 20 Gy to the contralateral side). Hence, the comparison of two RT doses within one patient could be performed effectively. This design controls risk factors and enables the detection of dose-dependent effects of RT between 50 and 70 Gy. Head and neck cancer patients without RT had similar risk factors and served as external control. Other limitations were the analysis of only one segment of the carotid artery and the semi-automatic image analysis. Additionally, the primary outcome—IMT—is a surrogate for vessel wall damage, but (unlike stenosis) IMT does not always correlate with a pathological state.

This study has clinical implications for planning and follow-up of head and neck cancer RT. Previous research established the risk of (ipsilateral) cerebrovascular events following head and neck RT [18, 26]. IMT increase precedes stenosis, plaque formation, and finally cerebrovascular events. Our study adds evidence to the correlation between high dose RT and an early increase of IMT. Therefore, radiotherapists should consider the additional toxicity of high doses for non-metastatic head and neck cancer. Recent de-escalation studies successfully omitted RT of the pathological pN0 neck and the contralateral clinical cN0 neck. These data indicate a paradigm shift away from standard bilateral neck RT towards a personalized approach with reduced toxicity [33–35]. However, dose reduction is impossible in metastatic cancer, as lymphatic drainage follows the course of the carotid artery. These patients should be informed about the risk of cerebrovascular damage. High-risk patients may be identified by IMT measurement during cancer aftercare. To confirm these claims, prospective studies with follow-up of IMT, stenosis, and cerebrovascular events are needed to enable interventional trials for effective prevention (e.g. antiplatelet drugs, statins, H_2_S) [36–39].

## Conclusion

Increased IMT and stenosis rates in unilaterally irradiated carotid arteries have been described previously [12, 17–19]. This study shows that high dose RT with a tumour side boost enhances toxicity to the carotid artery: IMT increased permanently within the first months. Therefore, planning of the RT boost should consider carotid artery toxicity. If neck metastases require RT with boost, IMT measurement is suitable for early detection of carotid artery damage.

## Data Availability

The datasets generated for this study are available on request to the corresponding author.

## Abbreviations

Gy: Gray
HNSCC: Head and neck squamous cell cancer
IMT: Intima-media thickness
RT: Radiotherapy
